# Lip-Reading: Advances and Unresolved Questions in a Key Communication Skill

**DOI:** 10.3390/audiolres15040089

**Published:** 2025-07-21

**Authors:** Martina Battista, Francesca Collesei, Eva Orzan, Marta Fantoni, Davide Bottari

**Affiliations:** 1 MoMiLab, IMT School for Advanced Studies Lucca, 55100 Lucca, Italy; martina.battista@imtlucca.it (M.B.); francesca.collesei@imtlucca.it (F.C.); 2 Burlo Garofolo Hospital, 34137 Trieste, Italy; eva.orzan@burlo.trieste.it (E.O.); marta.fantoni@burlo.trieste.it (M.F.)

**Keywords:** lip-reading, multimodal language processing, typical and atypical development, noisy environment, socio-cultural differences

## Abstract

Lip-reading, i.e., the ability to recognize speech using only visual cues, plays a fundamental role in audio-visual speech processing, intelligibility, and comprehension. This capacity is integral to language development and functioning; it emerges in early development, and it slowly evolves. By linking psycholinguistics, psychophysics, and neurophysiology, the present narrative review explores the development and significance of lip-reading across different stages of life, highlighting its role in human communication in both typical and atypical development, e.g., in the presence of hearing or language impairments. We examined how relying on lip-reading becomes crucial when communication occurs in noisy environments and, on the contrary, the impacts that visual barriers can have on speech perception. Finally, this review highlights individual differences and the role of cultural and social contexts for a better understanding of the visual counterpart of speech.

## 1. Introduction

Human communication is a fundamental cognitive ability that involves the exchange of dynamic signals, such as body posture, facial expressions, head and eye movements, gestures, and speech signals [1,2]. Optimal integration of the audio-visual speech components is crucial to communicate effectively in social contexts. The core topic of this review is “lip-reading”, considered as the ability of interpreting spoken language by observing the speaker’s facial and articulatory movements, without relying on auditory input. Indeed, while we mainly rely on auditory inputs for speech comprehension, it is also important to consider the visual counterpart of speech, particularly the movements and positions of the mouth, lips, and jaw during speech production. The visual aspects are commonly referred to as “visemes”, defined as a visually distinguishable unit in the visual domain corresponding to the phoneme in the auditory domain [3]. However, speech itself is intrinsically multimodal, involving the binding of auditory signals and visual information extracted from facial movements, including lips, which are coherently and dynamically modulated [4,5]. This multisensory integration confers significant advantages across various tasks. For instance, seeing a speaker’s articulatory movements can significantly benefit our understanding of the speech content [1].

The importance of this integration is substantiated by the fact that the ability to integrate visual and auditory information of speech signals emerges early in human development, ultimately suggesting a predisposition to bind these sensory inputs. Infants demonstrate this ability within minutes of birth by orienting their gaze toward speech signals [6]. In the first months of life, they show preferences for audio-visual presentations over purely visual ones [7]. They also exhibit preferences for congruent combinations of voice and speaker gender, as well as for matching vowel sounds and congruent articulatory gestures, becoming attuned to the phonetic statistics of their native language during childhood [8,9]. By 6 months, infants start detecting (in)congruences between the visual and the auditory component of speech, and around 8–9 months of life, they preferentially look at the speaker’s mouth; this gaze behavior is even stronger when hearing incongruent and non-native speech sounds. From a developmental perspective, this looking bias relates to linguistic and endogenous selective attention development. Also, it seems to improve both auditory perception and phonetic production and to predict subsequent auditory speech comprehension [10,11,12]. Thanks to these predispositions and early exposure to bimodal inputs, humans effortlessly integrate audio-visual speech stimuli, obtaining advantages in communication.

**Figure 1:**
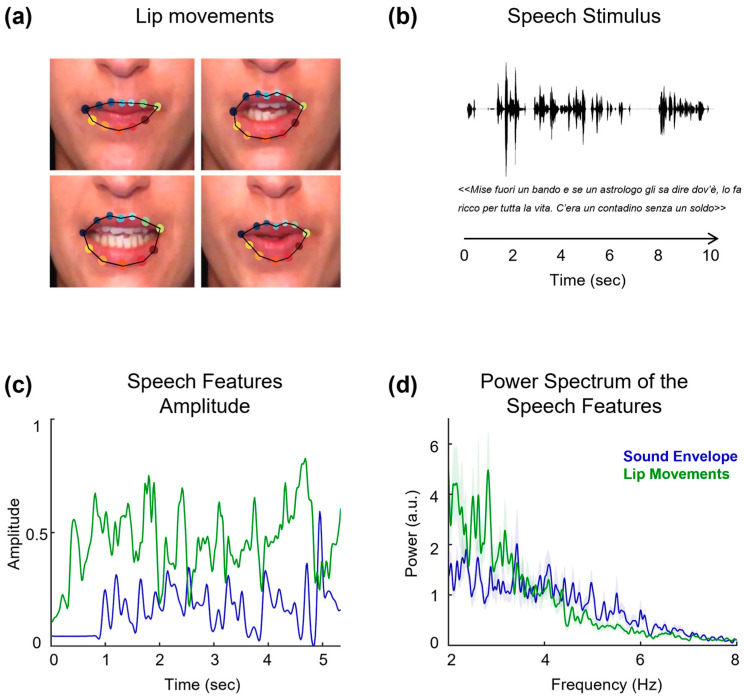
(**a**) Lip contour showing the target points used to extract a measure of lip movements over time in our speech stimuli. (**b**) Waveform of a 10 s audio speech stimulus with the corresponding transcript. (**c**) Examples of speech features dynamics: sound envelope (blue) and mouth area, indicating lip movement articulation (green). (**d**) Examples of power distribution for the speech envelope (blue) and lip movements (green) across the speech segments. This measure reflects the strength and variability of the sound envelope and lip movements over time.

To study the integration process of speech signals, researchers model features associated with the sound of speech and the dynamic movements of the mouth (Figure 1). For instance, the speech envelope represents the amplitude fluctuations of the speech signal over time, while the changes in the mouth area correspond to the temporal patterns of lip movement articulation. By comparing the patterns of these two features, it has been possible to investigate the degree of correlation between visual and auditory components of speech signals [5]. These studies revealed a strong temporal alignment between mouth opening and both the acoustic envelope and vocal tract resonance. Moreover, these signals exhibit modulations within the 2–7 Hz frequency range, with mouth movements consistently preceding vocal onset by 100 to 300 ms.

In everyday conversations, we are usually exposed to noisy environments that can degrade the auditory component of speech; thus, having access to complementary visual information compensates for underspecified auditory information, enhancing perception in challenging hearing conditions [13,14]. This mechanism arises from how visible articulators—such as lips, teeth, and tongue—align with the resonances of the vocal tract. These resonances continuously change in frequency, reflecting phonetic aspects of speech, including vowels, diphthongs, and consonant articulation [15]. Mismatches between these visual and auditory speech inputs can lead to perceptual illusions, such as the McGurk effect. For instance, when visual lip movements (e.g., the syllable “ga”) are coupled with incongruent but similar auditory stimuli (e.g., the syllable “ba”), observers often perceive a fused percept (e.g., the syllable “da”), highlighting the compelling influence of visual information on auditory perception [16]. Given its role in speech processing, lip-reading is crucial for increasing speech intelligibility in populations with hearing loss or hearing disturbances.

However, even in hearing populations and optimal listening conditions, visual speech can still provide an additional benefit, adding supplementary information [2,17]. This review aims to explore humans’ lip-reading ability from different perspectives. First, we will present a few tests that are commonly used to measure lip-reading skills and discuss how lip-reading ability changes across the lifespan (children, adults, and elders); then, we will stress the impact of cultural differences on lip-reading performance and highlight the importance of developing and using standardized tests for assessing lip-reading across diverse populations. Next, we will focus on the effects of atypical development on lip-reading ability (e.g., in the case of deafness and language impairments), shedding light on how different developmental trajectories can shape visual speech perception. Finally, we will highlight the role of lip-reading in noisy environments, e.g., “the cocktail party effect”, where background noise challenges auditory perception. We will also examine the detrimental effects of visual barriers, such as face masks, on speech perception, which prevent reliance on lip movements. Our aim was to provide a broad overview of the different perspectives involved in the study of lip-reading. This narrative review does not adhere to systematic review protocols. Following a thorough study of research articles on lip-reading, we selected works focusing on lip-reading assessment tests, typical and atypical development, noisy environments, socio-cultural factors, and the neural basis of lip-reading development.

## 2. Tests to Assess Lip-Reading Skills in Children, Adults, and Hearing-Impaired Individuals

The first expert who stressed the importance of designing tests for specifically assessing lip-reading skills was Utley, who designed the “Utley Lip-reading test” (see Table 1) [18]. Until then, teachers and clinicians evaluated individuals’ lip-reading ability subjectively.

Aiming to develop and implement a comprehensive lip-reading battery for assessing children’s visual word and sentence recognition abilities, Tye-Murray and colleagues [19] advocated the introduction of new measures. These tools were specifically designed to evaluate how children recognize words and sentences through silent-speech input only. Recognizing the limitations of existing measures for pediatric populations, the researchers introduced several novel assessments; in particular, the Tri-BAS (three “build-a-sentence” tests), the Illustrated Sentence Test (IST), the Gist Test, and the existing Children’s Audio-visual Enhancement Test (CAVET) (for all the tests see Table 1) created by Tye-Murray and Geers in 2001. Each of these tests serves a unique function and targets different aspects of visual language processing. The Tri-BAS uses a closed-set response format in which children are presented with a sentence frame (e.g., “The…watched the…”) and a matrix of pictures and need to select the two correct pictures that correspond to the target words in the sentence. The IST employs an open-set format with context-rich illustrations presented before a video clip of a speaker pronouncing the target sentence. Participants are asked to repeat the sentence for scoring.

In contrast, the Gist Test aims to evaluate participants’ ability to understand the overall meaning of sentences, or “gist”, rather than focusing on word-by-word recognition. Instead, the traditional test “CAVET” assesses visual word recognition in a carrier phrase format, in which participants are asked to repeat the final word of a phrase pronounced by a speaker in a video clip. Together, these tests form a robust framework for evaluating lip-reading ability in children. They allow clinicians and researchers to investigate not only the functioning of word and sentence recognition but also broader comprehension processes in visually presented speech.

Over the years, experts have designed standardized tests for adults, which are fundamental for distinguishing between atypical development characteristics, cultural differences, and individual experiences. For instance, in audio-visual (AV) contexts, Feld and Sommers [20] reported that even within a relatively uniform group, such as hearing college students, accuracy at lip-reading can easily fall within a range between 60% and 70%. At the same time, their auditory speech perception has higher scores, at least under favorable listening conditions.

Moreover, researchers have specifically developed tests aimed at assessing lip-reading skills in individuals with hearing deficits. For instance, the CUNY Sentence Test, created by Boothroyd et al. in 1985 [21], evaluates speech perception and is usually employed with individuals with hearing impairments, particularly post-lingually deafened cochlear implant users (see Table 1). The CUNY test has also been used to complement measurements in experimental settings involving electrophysiological assessments. For instance, Debener and colleagues [22] used the CUNY test and other standard clinical assessments to evaluate speech perception before and after cochlear implantation in deaf participants. They aimed to provide behavioral evidence supporting the electrophysiological investigation of lip-reading abilities. Studies employing fMRI have also shown a link between behavioral measures of silent speech perception and imaging data [23]. More recently, Plant and colleagues [24] have considered the CUNY test a possible assessment tool for long-term evaluation of a cochlear-implanted patient’s speech recognition performance.

### Practical Considerations, Limitations, and Future Directions

It is not yet possible to establish a ranking among the available lip-reading tests, as each is designed to address different assessment goals, populations, and methodological frameworks. For example, the Tri-BAS and CAVET are especially well-suited for assessing lip-reading in children thanks to their structured format and visual prompts. The IST offers advantages when a more open-set, contextually rich format is needed. Meanwhile, the CUNY Sentence Test has mainly been adopted for adults with hearing loss or cochlear implants.

Although all the above-mentioned tests remain invaluable tools in speech perception and lip-reading research, they present limitations. First, they often lack realism and ecological validity. By relying on isolated word lists, sometimes sentences, these tests do not mimic natural conversations. Furthermore, these tests often use a limited set of speakers, which might not account for the wide variety of accents, speech patterns, and articulation characterizing natural language production. As an example, a few researchers have tried to adapt some standard lip-reading sentence tests (mainly North American) to the linguistic variety of their country (e.g., New Zealand [25]) for potential clinical use in audiology.

Nevertheless, developing novel measures and metrics for assessing lip-reading ability would be necessary to provide more accurate assessments of individuals’ lip-reading skills that account for the naturalistic contexts in which everyday communication occurs. To ensure broader applicability, future assessments should also be standardized and culturally adapted, reflecting linguistic diversity and communication norms across different populations. Furthermore, they could be designed to explicitly map developmental trajectories by incorporating tests specifically dedicated to assessing children’s lip-reading ability as a function of their age and perceptual skills.

**Table 1:** The key characteristics of major lip-reading assessments, including their intended target groups, main strengths, limitations, and degree of ecological validity. It reflects the information reported in this review and serves exclusively as a summary of the reported information.

Test Name	Target Group	Strengths	Weaknesses	Ecological Validity
Utley Lip-reading Test	General (historically used for children)	First attempt to standardize lip-reading evaluation; raised awareness of the need for objective assessment tools	Outdated; subjective scoring; lacks developmental sensitivity	Low—Relies on isolated words/sentences
Tri-BAS (Build-a-Sentence)	Children	Structured closed-set format; uses picture aids; reduces cognitive demand; well-suited for younger population	Limited in assessing spontaneous or contextual comprehension	Moderate—Contextual clues from images, but lacks natural dialogue
Illustrated Sentence Test (IST)	Children	Open-set format with context-rich illustrations; encourages naturalistic sentence processing	Requires higher verbal output; may be harder for very young or language-delayed children	Moderate to High—Contextual imagery enhances realism
Gist Test	Children	Focuses on overall sentence meaning; useful for assessing higher-level comprehension	Less precise for word-level analysis; performance may vary due to the interpretive nature	Moderate—Emulates real-life gist-based understanding
CAVET (Children’s Audio-visual Enhancement Test)	Children	Simple carrier-phrase format; good for basic visual word recognition	Narrow focus on final-word repetition; limited sentence context	Low—Lacks conversational or contextual elements
CUNY Sentence Test	Adults with hearing loss or cochlear implants	Validated with clinical populations; used in research and clinical tracking; compatible with neuroimaging	Sensitive to subject fatigue; concentration-dependent; low realism	Low to Moderate—Uses full sentences but lacks conversational flow and speaker variability

## 3. The Development of Lip-Reading Abilities

Lip-reading has been mainly studied in two types of contexts: audiovisual contexts and visual-only contexts. In audiovisual contexts, both speech sounds and the visual information of the lips are present to test their interaction, while in visual contexts, there is no acoustic information. We will first focus on the factors influencing developmental trajectories in audiovisual contexts.

The variability in people’s reliance on lip-reading for processing speech is influenced by numerous factors, some of which could be age-related or dependent on individual differences such as experience, cultural differences, and typical or atypical development. Considering age-related factors, research has focused on how children [26,27], adults [20,26], and elders [20,28] rely on lip-reading, describing how this ability changes during different stages of life.

### 3.1. Infant Sensitivity to Visual Speech and Perceptual Narrowing

Visual exposure to the mouth is fundamental in infants’ early speech sensitivity and prepares them for speech production between 1 and 2 years of age [27]. To imitate sounds, babies need to learn how to shape their lips according to the sounds they hear, and watching a speaker’s mouth facilitates this learning process. Newborns can mimic adult mouth movements like sticking out the tongue or opening the mouth, which may be foundational for language acquisition [29]. Young children’s reliance on visual cues, especially lip movements, demonstrates the importance of mastering lip-reading as a human developmental milestone. Furthermore, it should be stressed that the way lip-reading develops at early stages of life is strictly intertwined with a critical characteristic of human development: perceptual narrowing, which refers to a developmental phenomenon observed in infants in early ontogeny, especially between 4 and 9 months of age [30]. Until around 6 months, most hearing infants are sensitive to a broad array of speech gestures, including those visible on the mouth, regardless of whether these gestures are part of their native language’s phonology. Then, in the second six months of life, infants undergo perceptual narrowing. That is, they show a decline in sensitivity to audiovisual distinctions that are not prevalent in their linguistic and social environments (e.g., non-native visual speech patterns and sounds), and they become more attuned to the phonetic structures of their primary language [31]. For instance, the speech sounds /v/ and /b/ are distinguishable in English but not in Castilian Spanish. Indeed, a seminal study [31] demonstrated that Spanish-exposed and English-exposed infants can distinguish these audiovisual speech elements up to about 6 months of age, demonstrating the ability to capture phonetic and articulatory changes beyond their native language. However, Spanish-exposed infants older than 6 months lose the capacity to distinguish between the audiovisual presentation of /v/ and /b/, whereas English-exposed infants retain this ability. Spanish children not exposed to English lose the ability to distinguish linguistic audiovisual correspondences that do not belong to their native language. These findings suggest that multimodal speech processing is integral to language development in the infant brain [32].

### 3.2. Language-Specific Tuning in Childhood

Several studies on developmental trajectories of lip-reading focused on how mastering this ability changes across childhood in young and older children. During childhood (ages 5–14), visual speech cues, such as lip movements, play a crucial role in helping children learn and process language effectively [19,33]. Indeed, effectively employing lip-reading contributes to children’s integration of auditory and visual speech information, as demonstrated in research studies on 5- to 8-year-old children’s vowel matching and the McGurk effect [33].

Research findings have shown that lip-reading improves with age. However, data are scarce, and the literature does not converge towards a uniform pattern of lip-reading development, showing heterogeneous and contrasting findings. Erdener and Burnham [33] highlighted that children substantially increase their lip-reading ability from 5 to 8 years old. Tye-Murray and colleagues [19] extended the development period for this function and observed significant improvements in lip-reading from 7 to 14 years of age. Notably, Tye-Murray’s evidence supports a protracted developmental trajectory of lip-reading ability in children and is consistent across four objective tests for assessing lip-reading ability.

In visual-only settings, researchers have investigated the role of visemes in lip-reading [34,35], the visual aspect of speech, and the movements and positions of the mouth, lips, and jaw during speech production. In a seminal work, Peymanfard and colleagues [35] showed that visemes are vital for developing lip-reading technological systems that are efficient at the visual recognition of linguistic cues. Indeed, as the authors stressed, a computational lip-reading model that focuses first on converting a video into visemes and then visemes into characters highly facilitates sentence decoding. By adding the viseme level as the intermediate level between individual sounds and the sentences, lip-reading systems better capture the complexity of language. Taken together, the presented findings on children’s lip-reading development emphasize the inherent predisposition for audiovisual integration, with a developmental trajectory extending for several years. However, while significant progress has been made in understanding the mechanisms of perceptual narrowing and the linguistic development of speech, including prosody and phonemes [36], our knowledge regarding the processing of lip movements remains limited and would require further research. A well-defined developmental trajectory of the ability to lip-read is not yet available. This gap in understanding is likely due to the need to implement specialized tests tailored for young children, as well as tests capable of effectively extracting and analyzing visemes.

### 3.3. Declining Lip-Reading Ability with Aging

Considering adults’ ability to read lip movements, most studies have focused on the changes occurring from young to older adults, especially concerning concomitant hearing loss [28]. Hearing adults with typical development can usually correctly integrate auditory and visual information [37]. This is partly due to the established linguistic background and reading skills acquired over the years. As hearing becomes less reliable with age, older adults may increasingly depend on lip-reading and are often encouraged to do so [37]. Nevertheless, the increased reliance on lip-reading does not imply better lip-reading ability but a decline with age [38], which is somewhat surprising for at least two reasons [20]. Firstly, older adults might find themselves increasingly dependent on visual speech perception due to age-related hearing impairments. Secondly, since age-related hearing loss progresses gradually, older adults can acquire progressively skills in encoding visual speech information over several years. This decline in lip-reading ability in older adults may be attributed to age-related impairments in working memory (WM) and information processing speed (PS), which are crucial cognitive abilities for effective lip-reading [20]. In particular, Feld and Sommers [20] focused on the role of WM as a cognitive ability, either verbal, recalling the denomination presented items, or spatial, recalling the locations of presented information. PS was employed to derive a reaction time score in three tasks based on the accuracy and speed of response. The authors showed that impairments in these cognitive abilities (WM and PS) are correlated with impoverished lip-reading [20]. That is, cognitive decline, which can precede or accompany measurable hearing loss, further complicates the issue [39]. Consequently, while lip-reading can support hearing loss, it has been proven to be highly difficult for elders, and it may not entirely offset the combined effects of diminished hearing and cognitive abilities in older adults.

## 4. Culture and Lip-Reading

The ability to integrate auditory and visual speech cues is not only shaped by individual development but also by cultural and linguistic context. Recent research has increasingly emphasized that both language structure and culturally influenced perceptual habits contribute to differences in how individuals process audio-visual speech. These variations begin early in life and continue to influence lip-reading performance into adulthood, highlighting the importance of considering cultural context in both research and clinical assessment.

### 4.1. Developmental and Cultural Variations in Lip-Reading Ability

Considering cross-cultural differences, various studies highlighted how linguistic and cultural factors shape audio-visual speech processing. Sekiyama, Burnham [40], Hisanaga and colleagues [41] highlighted the impact of cultural differences on lip-reading abilities, leveraging the abilities of Japanese and English people. The authors revealed that native Japanese speakers are less subject to visual influence in speech perception compared to native English speakers. Interestingly, the inter-language difference in visual influence is already evident at around 8 years of age, with English children showing an enhanced visual influence compared to Japanese children [40]. These cultural differences could be due to language [42] and gaze bias [41]. The first refers to different language experiences. Indeed, language experience plays a significant role in auditory–visual speech perception [42]. While for English children, the use of visual information increases over age, indicating a developmental promotion of audio-visual integration, Japanese children showed early auditory superiority in accuracy, which may lead to greater auditory-dependent processing in audio-visual speech perception at later stages [40]. The second bias refers to the cultural differences in gaze patterns [41,43]. That is, native English speakers (ESs) tend to show a gaze bias towards the mouth, particularly before the onset of auditory speech.

In contrast, native Japanese speakers (JSs) and Chinese speakers do not exhibit the same bias and instead focus more on the eyes and nose [43]. This difference in visual attention is suggested to be influenced by linguistic and cultural factors, indicating that Eastern and Western individuals may prioritize different facial regions when processing audio-visual information [41,43].

### 4.2. Tonal Languages and Reduced Visual Influences

Another example of Eastern and Western countries’ cross-cultural variation in audio-visual speech processing comes from studies examining tonal languages. Research has shown that native speakers of tonal languages may rely more heavily on auditory cues than visual cues during speech perception, due to the functional load carried by pitch in distinguishing word meanings [44]. For instance, Burnham and Lau [44] found that Mandarin-speaking adults demonstrated reduced susceptibility to the McGurk effect (thus to the mismatch between visual and auditory speech) compared to English speakers, suggesting a diminished reliance on visual speech information. This pattern was also observed in children, with Mandarin-speaking children showing weaker visual influence in audio-visual tasks than their English-speaking counterparts. Therefore, the linguistic demands of tone languages may condition listeners to prioritize auditory input, potentially limiting the developmental emphasis on visual information for speech processing.

All these findings support the idea of a complex interaction between culture, multimodal language processing, structural features of a language, and visual exploration in shaping lip-reading abilities across different populations. However, a comprehensive understanding of the role of cultural differences in how lip-reading develops and functions is far from being achieved.

Future research should aim to explore these cultural and linguistic differences using standardized, ecologically valid, and culturally adapted assessments. Longitudinal and cross-linguistic studies, particularly those involving children, will be key to identifying how these influences emerge over time and how they may inform both clinical practice and the development of inclusive, globally relevant speech perception tools.

## 5. Lip-Reading Ability in Atypical Development

The study of atypical development is of fundamental importance to better comprehend the neural mechanisms underlying lip-reading and the behavioral outcomes deriving from this ability. Atypical development refers to developmental trajectories deviating from the typical or expected progression of milestones in one or more domains [45]. It can manifest as delay, deviation, dissociation, or regression compared to what is commonly observed in typically developing children [45]. Studies on populations with atypical development revealed crucial differences from typical development in the role of early sensory and cognitive processes [46]. Given the scope of the present review, we will first focus on studies concerning hearing impairments and deprivation since lip-reading may represent a distinct adaptation to sensory loss [47,48]. Then, we will present two cases of atypical development in the linguistic domain: dyslexia [37] and specific language impairments (SLI) [46] and their relationship with lip-reading.

### 5.1. Lip-Reading in Hearing-Impaired and Cochlear-Implanted Individuals

Extensive research has been conducted on the role of lip-reading in hearing-impaired individuals with or without hearing aids [49,50]. It is commonly claimed that deaf individuals rely more on lip-reading than hearing controls due to some plastic adaptation for auditory loss compensation. However, it is essential to distinguish between deaf individuals without and with hearing aids, since how lip-reading is adopted differs greatly among these individuals. The first group of profoundly deaf people relies only on visual information from the lips, hands, and body gestures (sign language) to understand language [17,47]. Therefore, lip-reading is crucial for communicating with others, especially in contexts of interaction with the hearing community where no sign language is commonly shared [51]. For the latter group, a further subcategorization is needed between hearing-impaired individuals with some degree of hearing loss who might be wearing a prosthesis to try to compensate for this loss and deaf people with cochlear implants. In this case, hearing aids, such as prostheses and cochlear implants, allow deaf people to access acoustic information, albeit with some degree of variability compared to hearing people. In studies about language learning and cochlear-implanted children (ages: 3–18), lip-reading has been demonstrated to be a primary means to integrate audiovisual information, such as learning about the phonological structure of spoken language [52]. Indeed, while lip-reading provides information about specific phonological contrasts (e.g., place of articulation) that are conveyed visually, the cochlear implants allow to capture other phonological information such as nasality and voicing [46]. Therefore, combining auditory information from hearing aids and visual information from lip-reading allows deaf individuals to overcome limitations on unimodal phonological representations, strengthening people’s acquisition of oral language [46].

Nonetheless, as for hearing people, profoundly deaf individuals, and people with hearing aids, the development of lip-reading ability is strictly related to a combination of experience, language adaptations, and visual sensitivity.

### 5.2. Lip-Reading Abilities in Individuals with Dyslexia

Considering other instances of atypical development and lip-reading, a language impairment that has been extensively investigated is dyslexia. Some studies have shown that dyslexic individuals may struggle with lip-reading due to potential deficits in establishing and maintaining sufficient phonological representations [37]. In the study by Mohammed and colleagues, considering the speechreading measures (a set of tests to identify words, sentences, stories, and minimal pairs), the deaf group significantly outperformed the hearing control group, who outperformed the dyslexic group. This indicates that the dyslexic group has difficulty visually processing and recognizing speech’s sounds and phonetic components [37]. Yet, other studies found that dyslexic individuals with lower phonological awareness scored higher on lip-reading, thus showing that lip-reading may serve as a compensatory mechanism to help them cope with difficulties in auditory speech processing [53]. Overall, the evidence points to a complex relationship where some dyslexic individuals may develop stronger lip-reading skills as a strategy to compensate for their phonological processing deficits.

In contrast, others may struggle with lip-reading altogether. This variability, once again, may be due to the different factors that contribute to the individual development of this ability. Nonetheless, especially in scholastic contexts, lip-reading assessment tests should be employed to evaluate the proficiency of dyslexic children in lip-reading. This evaluation can support the development of strategies to improve their phonological awareness, which is crucial for identifying and interpreting speech sounds.

### 5.3. Visual Speech Processing in Children with Specific Language Impairments (SLI)

Another example is the case of children or individuals with specific language impairments (SLI), who are poorer lip-readers compared to typically developing people (as an example, they can show reduced correct responses associated with lip-reading from 60% of controls to 42% [46]). SLI refers to a condition where a child experiences delays in both expressive and receptive language skills, particularly in phonological processing, which can lead to difficulties in understanding spoken language. The key finding by Heikkilä [46] was that phonological processing deficits in SLI were shown to extend to the perception of visual speech, further suggesting that problems in phonological processing may contribute to poor lip-reading skills in children with language impairments (ages: 7–10). Furthermore, a seminal study by Knowland and colleagues [54] assessed the ability of children with language impairments (ages: 5–11) to use visual speech cues from the talking face during tasks involving speech-in-noise and silent speechreading. The findings highlight that although visual cues are important to enhance speech perception, especially in challenging listening conditions, children with language impairments are worse at lip-reading (e.g., single words task: 48%) than their typically developing peers (60%) [54].

All these findings in atypical development highlight the fundamental interplay between sensory development, cognitive processes, and atypical developmental trajectories in shaping lip-reading skills across different populations. Indeed, in discussing these three instances of atypical development (deafness, dyslexia, and SLI), lip-reading emerges as a key and essential feature to enhance audiovisual speech perception, and, more broadly, communication. Furthermore, it is important to consider the implications of these types of studies on educational practices. Tailoring strategies to teach how to integrate visual speech cues could significantly support language development in young students with hearing impairments or linguistic deficits. Especially considering that school contexts are often chaotic and noisy environments where the auditory input could be easily degraded. In this respect, a fundamental approach to investigate the advantages of lip-reading abilities and the relationship between visual and auditory cues in speech processing has been provided by studies in which visual or auditory noise is present and manipulated.

## 6. Lip-Reading in Noisy Environments

In noisy situations, humans greatly benefit from receiving multisensory speech signals to enhance comprehension. In particular, the ability to extrapolate and efficiently process lip-reading information becomes crucial, even for hearing individuals [42]. A common noisy scenario that characterizes human interaction is represented by the “cocktail party” [55]. The “cocktail party problem”, first described by Cherry [55], illustrates the challenge of segregating and focusing on an auditory signal of interest, such as a speech signal, from a mixture of overlapping sounds [56]. Cherry’s work showed that simultaneous exposure to two messages prevents meaningful comprehension unless their contents are distinctly separate. In such environments, attention and perceptual strategies are continuously adjusted by the listeners, who rely on ongoing sensory cues to segregate the speech signal of interest from the interfering one, acting in a process defined as auditory stream segregation [57].

In such contexts, resonance in mid and high frequencies is often distorted, and lip-reading plays an essential role [15]. Indeed, by extracting information from lip movements, humans are able to withstand an additional 4–6 dB of noise while maintaining equivalent performance as when they are solely relying on auditory information [58,59]. A seminal study by Sumby and Pollack [13] was among the first to investigate the contribution of visual cues to speech intelligibility in noisy environments. They hypothesized that visual cues substantially enhance speech intelligibility, particularly at lower signal-to-noise ratios (SNRs). Their findings showed that combined audio-visual presentations significantly outperform auditory-only presentations in speech intelligibility gain, with the most pronounced benefits occurring at lower SNRs. For instance, at an SNR of −30 dB, visual cues improved intelligibility by up to 80% for small vocabularies. Substantial gains were still observed at −18 dB and −12 dB, while at 0 dB and above, the benefit of visual input diminished due to already high auditory intelligibility.

This underlines the critical role of visual cues in enhancing speech perception in high-noise conditions [13]. While early behavioral studies have confirmed these results [60,61,62,63], a more recent work [14] challenges previous findings, stressing the importance of employing a large set of stimuli and assessing the audio-visual gain across different SNRs. Results of this study showed that the multisensory speech system is finely tuned to maximize integration efficiency at intermediate SNR levels, where neither visual nor auditory input alone is optimal. This finding stresses the adaptive nature of sensory integration in speech processing in noisy environments, highlighting the benefits of relying on visual and auditory cues for effective communication. At these intermediate levels, speech recognition enhancement through multisensory means is substantial, resulting in more than a threefold improvement compared to relying on auditory input alone. Specifically, they observed the peak of audio-visual benefit at −12 dB SNR, with a word recognition improvement of ~45%; however, audio-visual gain was still evident at even −24 dB SNR, when speech was hardly understandable, though to a lesser extent. These results confirm a “special zone” of maximum benefit of AV integration at a specific noise level [14].

Shahin and colleagues [64] showed that visual cues from lip movements help listeners better segment and identify phonetic speech cues. This leads to improved comprehension and reduced cognitive load during speech perception tasks [64].

To further understand the improvement given by lip-reading to speech intelligibility in noisy contexts, the COVID-19 pandemic provided a unique natural model. Indeed, since the pandemic, researchers have had the opportunity to investigate the contributions of visual and auditory cues to audio-visual speech processing due to the widespread use of face masks. Face masks create both a visual barrier by obscuring lip movements and an auditory filter by altering speech sounds [65]. Recent studies have shown that face masks significantly worsen speech intelligibility even in moderately noisy environments [66]. Additionally, informational noise, such as multi-talker babble, poses a greater challenge to listeners than energetic noise, like speech-shaped noise (SSN), indicating that social environments may further complicate communication [67]. In this regard, Giovanelli and colleagues [68] investigated the impact of face masks on speech comprehension during multi-talker video calls. They found that obscuring the speaker behind the screen or covering lips with face masks can negatively impact performance, confidence, effort, and metacognitive monitoring during speech processing [69].

## 7. The Neural Basis of Lip-Reading

In recent years, new approaches have been developed to investigate how the brain tracks lip movement in combination with speech input or silence. These approaches leverage how the brain synchronizes with external input. To reduce variability and create stable sensory representations, neural oscillations tend to align with the timing of relevant input streams. This process adjusts the strength of sensory input, enhancing or diminishing information as a function of the oscillations’ phase. This mechanism is so widespread that it has been shown that various sensory stimuli, such as visual, auditory, and tactile, can entrain neural activity.

### 7.1. Neural Tracking and Integration of Visual Speech

Neural tracking (or neural entrainment) refers to the mechanism through which neural oscillations synchronize their activity with the temporal properties of sensory input [70,71,72]. Thus, by modeling features of continuous input, such as speech, it is possible to investigate to what extent the brain tracks and elaborates their properties.

Several studies have shown that visual cues significantly enhance the neural tracking of the auditory cortex to the speech envelope in response to audio-visual attended stimuli compared to unattended ones [1,66,67]. Notably, this enhancement is reduced in the absence of visual cues, such as in tasks solely relying on auditory stimuli. Along these lines, Park and colleagues [2] showed that lip movements entrain the low-frequency oscillations in the observers’ brains, enhancing speech intelligibility. This effect was most pronounced in conditions where audio-visual inputs were congruent, indicating that lip movements provide critical temporal cues that help the brain decode speech more efficiently [2]. Most interestingly, two studies [70,71] have shown that from silent lip-reading signals, the brain can form a coarse auditory speech representation in early auditory cortices. Their findings suggest a specific oscillatory mechanism: Visual observation of lip movements initially influences neuronal activity in early visual cortices at frequencies that align with the articulatory patterns of the lips (>1 Hz). The right angular gyrus contributes to extracting the slower features of these lip movements. It maps them to corresponding speech sound features, which are then relayed to the auditory cortices, potentially aiding speech parsing [73]. Moreover, recent findings have also shown that the brain specifically entrains to higher-level, visual representations of linguistic units, the visemes, in a distinctive manner compared to lip movements [69]. These results provided evidence that the visual system generates a specialized representation of speech that aligns closely with categorical linguistic features.

Interactions between visual and auditory neural representations have also been studied. Jaha and colleagues [57] explored the role of visual cues in discerning relevant speech in a cocktail party experiment. They revealed that visual networks facilitate comprehension by suppressing auditory cortex responses to irrelevant sounds, highlighting the interaction between visual and auditory processing when hearing is less reliable due to noise [57].

### 7.2. Impact of Face Masks on Neural Processing

Face masks, while behaviorally disruptive to speech perception, also impair neural tracking of speech. From a neurophysiological perspective, Fantoni and colleagues [65] investigated how the brain synchronize to speech signals in the presence of face masks and multi-talker noise, using the electroencephalography (EEG)—a direct and non-invasive measurement of brain’s electrical activity through electrodes placed on the scalp that allow to track the synchronization of neural responses with millisecond precision. They found that face masks abolish lip tracking and detrimentally affect neural tracking of the speech envelope. In particular, the absence of visual input associated with lip movements was found to alter the neural tracking of speech envelope at the early stages of processing, even in the presence of clear audio, suggesting the lack of the facilitatory effect of speech processing associated with lip-reading. Instead, listening to a speaker wearing a face mask, hiding the mouth, and degrading the acoustic signals (the auditory filter exerted by the mask) altered the neural tracking of the envelope also at later stages, revealing the distinct effects of visual and auditory barriers on different stages of speech processing. Along these lines, Haider et al. [74] investigated which aspects of speech processing are affected by the occlusion of the mouth area by means of face masks. They employed the magnetoencephalography (MEG)—a non-invasive neuroimaging technique that records magnetic fields generated by the electrical activity of neurons. Their results showed that the decoding of naturalistic speech is altered at both low-level acoustic features—speech envelope, pitch, and formants—and at higher-level segmentation features of speech, such as phonemes and word onsets. The latter shows a specific impairment in neural speech tracking in challenging listening conditions induced by a distractor speaker [74]. In subsequent work, they found that the negative effect of face masks on speech tracking and speech comprehension in general is not determined by the acoustic degradation of speech; it is instead specifically related to the absence of visual cues [75].

### 7.3. Developmental Cortical Mapping of Visual Speech

While these findings emphasize the critical role of visual input in adult speech processing, much less is known about how these mechanisms emerge and evolve during infancy and early childhood. Behavioral studies have shown that visual speech discrimination appears to follow a developmental trajectory that aligns with auditory speech processing, particularly in terms of perceptual attunement. In the first months of life, infants can detect congruence between visual articulations and the auditory speech counterparts and learn to use silent visual speech to discriminate between languages. However, by around 8 months of age, they begin to show an attunement to their native languages, which happens in parallel with a gain in visual attention to talking faces, likely related to increased ability to read visemes [76]. The cortical mapping of visual speech during development has been investigated less, leading to a few mixed findings. For instance, one study by Dopierala and colleagues [77] used functional Near-Infrared Spectroscopy (fNIRS)—a non-invasive technique that measures blood oxygenation changes in the brain—to investigate the putative role of the temporal visual speech area (TVSA) in processing visemes. While this area is known to contribute to viseme cortical representations in adults, they investigated cortical responses to silent visual speech and non-speech mouth movements in infants aged 5 and 10 months to investigate how these representations develop. At 5 months, both stimuli (speech and non-speech mouth movements) elicited similar fronto-temporal cortical activations. This suggests that infants at this age do not yet differentiate between linguistic and non-linguistic mouth movements. By 10 months, infants started having specific cortical responses, indicating a developing specificity for visual speech. Thus, visual speech starts to be represented for general mouth movements and becomes specific to speech articulation only later in life [77].

A few studies have also explored the neural tracking of visual cues during continuous speech and how this process evolves throughout development. Tan and colleagues [78] recorded EEG and eye-tracking data from 5-month-olds, 4-year-old children, and adults while listening to speech in auditory-only, visual-only, and audio-visual conditions. Their results showed that both 5-month-old infants and adults showed more accurate speech tracking in audio-visual conditions compared to auditory and visual conditions alone, suggesting a benefit in cortical tracking of the speech envelope in the bimodal condition [78]. A recent study investigated whether lip movement tracking provides a gain in neural speech tracking in 10-month-old infants compared to auditory-only conditions. While they observed a neural tracking of continuous speech within the 1–10 Hz frequency range, no advantages were found in the audiovisual condition, suggesting that visual speech cues did not yet modulate neural tracking at this stage of development [79].

## 8. Conclusions

Lip-reading is an essential ability in human speech processing. It emerges from a very young age when infants rely on detecting lip movements for bootstrapping language learning, and it gradually develops. However, factors other than age affect this function, revealing its experience-dependent nature. For instance, culture, as well as typical and atypical developmental pathways of auditory and, in general, language functions, can change the processing of the visual counterpart of speech. While in the past, research was confined to behavioral assessments, novel analytical tools have recently enabled the initial mapping of the neural correlates of lip-reading functions, both in silence and when combined with speech sounds. However, the field is new. The brain’s processing of sounds along the language hierarchy has been extensively studied, but the same cannot be said for lip-reading.

Taken together, both behavioral and neurophysiological findings presented here offer insights for future research directions. Concerning the developmental aspects of lip-reading, there is still a lack of standardized measures that can reliably assess lip-reading abilities across ages, languages, and populations. Specifically, longitudinal studies from infancy through adulthood would be needed to clarify the contributions of sensory and language experience for characterizing the developmental trajectory of lip-reading. Cross-cultural and cross-linguistic research would also be required to better understand how language structure and cultural norms, such as audio-visual exposure, might impact visual speech processing. Moreover, studies in atypical populations, such as individuals with hearing loss, autism spectrum disorder, or language impairments, have the potential to offer unique insights about the flexibilities of the systems underpinning lip-reading.

By integrating standardized cross-cultural behavioral approaches and novel methods for estimating the neural correlates of naturalistic stimuli, such as continuous speech, future research will provide valuable insights for the understanding of the multifaceted nature of lip-reading. An elusive but fundamental human ability with a slow-paced development and with a relevance that discloses when the sounds of speech are not enough to be communicated.

## Data Availability

No new data were created or analyzed in this study. Data sharing is not applicable to this article.

## Abbreviations

The following abbreviations are used in this manuscript:

AV	Audiovisual
IST	Illustrated Sentence Test
WM	Working memory
PS	Processing speed
CAVET	Children’s Audio-visual Enhancement Test
Tri-BAS	3 × 3 Build-A-Sentence
CUNY	City University of New York
Ess	English speakers
JSs	Japanese speakers
SLI	Specific language impairments
SNRs	Signal-to-noise ratios
SSN	Speech-shaped noise
MEG	Magnetoencephalography
EEG	Electroencephalography
fNIRS	functional Near-infrared spectroscopy
TVSA	Temporal visual speech area
